# Dysregulation of Mesenchymal Stromal Cell Antioxidant Responses in Progressive Multiple Sclerosis

**DOI:** 10.1002/sctm.18-0045

**Published:** 2018-07-31

**Authors:** Juliana Redondo, Pamela Sarkar, Kevin Kemp, Kate J Heesom, Alastair Wilkins, Neil J Scolding, Claire M Rice

**Affiliations:** ^1^ Clinical Neuroscience, Translational Health Sciences University of Bristol Bristol United Kingdom; ^2^ Proteomics Facility University of Bristol Bristol United Kingdom

**Keywords:** MSC, Antioxidants, Multiple sclerosis, Nitrosative stress, Cell therapy

## Abstract

The potential of autologous cell‐based therapies including those using multipotent mesenchymal stromal cells (MSCs) is being investigated for multiple sclerosis (MS) and other neurological conditions. However, the phenotype of MSC in neurological diseases has not been fully characterized. We have previously shown that MSC isolated from patients with progressive MS (MS‐MSC) have reduced expansion potential, premature senescence, and reduced neuroprotective potential in vitro. In view of the role of antioxidants in ageing and neuroprotection, we examined the antioxidant capacity of MS‐MSC demonstrating that MS‐MSC secretion of antioxidants superoxide dismutase 1 (SOD1) and glutathione S‐transferase P (GSTP) is reduced and correlates negatively with the duration of progressive phase of MS. We confirmed reduced expression of SOD1 and GSTP by MS‐MSC along with reduced activity of SOD and GST and, to examine the antioxidant capacity of MS‐MSC under conditions of nitrosative stress, we established an in vitro cell survival assay using nitric oxide‐induced cell death. MS‐MSC displayed differential susceptibility to nitrosative stress with accelerated senescence and greater decline in expression of SOD1 and GSTP in keeping with reduced expression of master regulators of antioxidant responses nuclear factor erythroid 2‐related factor 2 and peroxisome proliferator‐activated receptor gamma coactivator 1‐α. Our results are compatible with dysregulation of antioxidant responses in MS‐MSC and have significant implications for development of autologous MSC‐based therapies for MS, optimization of which may require that these functional deficits are reversed. Furthermore, improved understanding of the underlying mechanisms may yield novel insights into MS pathophysiology and biomarker identification. Stem Cells Translational Medicine *2018;7:748–758*

**Significance Statement**

The potential of autologous mesenchymal stromal cell‐based therapy for multiple sclerosis (MS) and other neurological diseases is currently being explored in clinical trials although the effects of disease on mesenchymal stromal cell phenotype and function have not been extensively examined. Results of this study show that mesenchymal stromal cells isolated from patients with progressive MS have increased susceptibility to nitrosative stress and reduced expression, activity, and secretion of key antioxidants. These findings have important implications not only for the development of autologous mesenchymal stromal cell therapy for MS but also for other conditions in which mesenchymal stromal cell function has not been fully characterized. In addition, the potential contribution of mesenchymal stromal cell dysfunction to the pathophysiology of progressive MS and/or its comorbidities should be explored further.

## Introduction

Reactive oxygen and nitrogen species (ROS/RNS) are by‐products of normal aerobic metabolism and are involved in the complex regulation of several signaling pathways including cell proliferation, survival, and inflammation 1, 2, 3, 4, 5, 6, 7, 8, 9. Imbalance between ROS/RNS and antioxidant function results in oxidative/nitrosative stress that contributes to pathology in a range of clinical contexts including ageing and multiple sclerosis (MS) 10, 11, 12, 13.

MS is an inflammatory demyelinating and neurodegenerative disease of the central nervous system, and progressive forms of MS are characterized by a relentless accumulation of neurological disability over time for which effective treatment is a major unmet clinical need. Mesenchymal stromal cells (MSCs) have a range of properties of relevance to cell therapy for MS including anti‐inflammatory, immunomodulatory, and antioxidant paracrine activity 14. Given the wealth of preclinical data demonstrating amelioration of disease and their favorable safety profile, there has been rapid clinical translation of autologous MSC‐based cell therapy for MS 15. However, studies have shown that MSC function changes with age and chronic exposure to a pro‐inflammatory environment 9, 16 and few data are available regarding MSC function in MS.

We have recently demonstrated that MSC isolated from patients with progressive MS (MS‐MSC) have reduced ex vivo proliferation and clonogenic potential*,* premature senescence, and accelerated shortening of telomere terminal restriction fragments 17. We have also shown that the MS‐MSC secretome has reduced in vitro neuroprotective potential 18. Recently, others have demonstrated abnormalities in MSC isolated from patients with progressive supranuclear palsy (PSP) 19. These findings add to the growing body of literature documenting altered MSC function in disease states, and the role of MSC in their pathogenesis, and/or development of associated comorbidities 20, is now under investigation in a range of clinical contexts including ageing syndromes 21, 22, metabolic syndrome 23, diabetes 24, 25, rheumatoid arthritis 26, and systemic lupus erythematosus 27.

Given that nitrosative stress has been implicated in the pathogenesis of ageing 10, neurodegeneration 2, and MS 11, 12, we sought to examine the antioxidant capacity of MS‐MSC and their susceptibility to nitric oxide‐induced cell death as determined by exposure to DETANONOate, a nitric oxide donor.

## Materials and Methods

### Study Cohort

MSC were isolated with appropriate consent from bone marrow samples from individuals undergoing elective total hip replacement surgery (control MSC [C‐MSC]; UK Research Ethics Committee [REC] 10/H102/69) and patients with progressive MS (MS‐MSC) participating in the ACTiMuS (Assessment of Bone Marrow‐Derived Cellular Therapy in Progressive Multiple Sclerosis, NCT01815632, REC 12/SW/0358) trial 17. The clinical details of control and MS subjects (sex, age, classification of MS, duration of disease progression, and exposure to disease modifying therapy [DMT]) are presented as Supporting Information (Table S1) together with the inclusion and exclusion criteria for the ACTiMuS trial (Supporting Information Table S2). In summary, patients with either primary or secondary MS with an Expanded Disability Status Scale 28 of 4–6 were eligible for the study if they were systemically well despite a clear history of disease progression in the preceding year during which time they must not have been on DMT for MS.

The control cohort were older; median age of control subjects 58.5 years old (7 males and 7 females) and median age of MS patients 53 years old (13 males and 16 females; unpaired *t* test *p* = .003). There was no sex bias between the cohorts (*p* = .772) and an independent effect attributable to birth sex was not observed in analyses. The control cohort had not been exposed to immunomodulatory therapy in the past. None of the 13 patients with primary progressive MS had been exposed to immunomodulatory therapy or DMT. Of those with secondary progressive MS (*n* = 16), eight had previously been treated with DMT (50%), and in all cases, treatment had been discontinued >1 year prior to collection of marrow in keeping with the entry criteria for the ACTiMuS trial. Not all samples were available for all experiments; the number of biological replicates is specified in each experiment individually and details regarding the cohort and which samples were used for each analysis are presented as Supporting Information. There was no significant association between age and duration of disease progression in the MS cohort. Although there were insufficient patients with a history of exposure to DMT included in the experiments to perform regression analysis, there was no difference between the cohorts with primary and secondary progressive disease in any of the analyses.

### Isolation of Bone Marrow‐Derived MSC

MSC from control and MS marrow were isolated using density gradient centrifugation and seeded in 25 cm^2^ flasks (passage 0) with medium consisting of low glucose Dulbecco's modified Eagle's medium (DMEM) (Sigma USA) with 10% foetal bovine serum (FBS) selected for the growth of MSC (Gibco USA) and 1% Penicillin and Streptomycin (Sigma). At approximately 70% confluence, plastic‐adherent cells were detached using 0.25% trypsin (Sigma) and reseeded at 2.5 × 10^5^ cells per 75 cm^2^ flask (passage 1). To ensure isolated MSC conformed to international defining criteria 29, cell surface phenotype and mesenchymal differentiation potential were examined as previously reported 30. For all experiments, both control (C‐MSC) and MS‐MSC were matched for passage number (either at passage 3 [p3] or passage 4 [p4]) to avoid potentially confounding effects.

### MSC‐Conditioned Medium

Confluent MSC cultures, at either p3 or p4, were washed twice in DMEM and cultured for 24 hours in minimal medium (MIN), consisting of DMEM without serum supplemented with 1% insulin‐free Sato (containing 100 μg/ml bovine serum albumin [BSA], 100 μg/ml transferrin, 0.06 μg/ml progesterone, 16 μg/ml putrescine, 0.04 μg/ml selenite, 0.04 μg/ml thyroxine, and 0.04 μg/ml triiodothryonine), 1% Penicillin and Streptomycin, and 0.5% l‐glutamine. Conditioned media (CM) was collected, filtered, and frozen at −80°C for future experiments.

### Proteomics

Protein was extracted from MSC using mirVana PARIS RNA and protein purification kit (Life Technologies USA), following which samples were incubated on ice for 30 minutes and then centrifuged at 10,000*g* for 10 minutes. The supernatant was collected and stored at −80°C. Protein content was determined using the Qubit Fluorometer and Quant‐iT Protein assay kit (Invitrogen USA) according to manufacturer's instructions and diluted to 2 mg/ml.

Liquid chromatography‐tandem mass spectrometry (LC‐MSMS) of C‐MSC and MS‐MSC conditioned medium was performed by the University of Bristol Proteomics Facility using a previously described protocol for tandem mass tagging (Thermo Fisher Scientific USA) coupled to liquid chromatography‐mass spectrometry 31. Values were normalized to a randomly selected control.

### Enzyme‐Linked Immunosorbent Assay

Ready to use sandwich enzyme‐linked immunosorbent assay (ELISA) assay for superoxide dismutase 1 (SOD1) (Affymetrix USA) and Glutathione S‐Transferase Pi 1 (GSTP1) (Cusabio USA) were performed on 1:1 diluted CM from MS and control MSC according to manufacturer's instructions. A standard curve was prepared, and absorbance read on a spectrophotometer at 450 nm. Values were interpolated into the curve and multiplied by the dilution factor to obtain the final concentration.

### DETANONOate Toxicity Assay

Prior experiments have demonstrated a linear relationship between methylthiazolyldiphenyl‐tetrazolium bromide (MTT) signal and MSC live cell number (data not shown). A stock solution (50 mM in 10 mM NaOH) of (Z)‐1‐(2‐([minoethyl]‐*N*‐[2‐ammonioethyl]amino)daizen‐1‐ium‐1,2‐diolate (DETANONOate; Enzo Life Sciences UK) was prepared immediately before use. Cells were seeded at 1 × 10^4^ cells per well in a 96‐well plate. Postadherence, basal culture medium was removed and cells washed in DMEM before addition of MIN or MIN with DETANONOate at the relevant concentration for 24 hours. Subsequently, medium was removed and warm phosphate buffered saline containing 10% FBS and 1 mg/ml of MTT added. After incubation for 3 hours at 37°C, the solution was aspirated and plate left to dry before 50 μl per well dimethyl sulfoxide (Sigma) was added and absorbance read at 570 nm following dissolving of formazan. Optical density (OD) values from a single patient were normalized to its own MIN value to allow comparison between samples from different patients.

### Bromodeoxyuridine Cell Proliferation Assay

MSC were seeded at 1 × 10^4^ cells per well in a 96 well plate. DETANONOate and bromodeoxyuridine (BrdU) (Millipore UK) were added following cell adherence for 24 hours. Cells were subsequently fixed, washed, and incubated with anti‐BrdU monoclonal antibody for 1 hour according to manufacturer's instructions. After the addition of the peroxidase‐conjugated secondary antibody, substrate, and stop solution, the OD signal was measured using a spectrophotometer with a wavelength of 450 nm.

### Senescence‐Associated β‐Galactosidase Staining

C‐MSC and MS‐MSC were seeded at 5 × 10^4^ cells per 35 mm wells, treated with DETANONOate 0.6 mM for 24 hours and stained with the senescence‐associated β‐galactosidase (SA‐β‐gal) kit (Cell Signaling Technology USA).

### Immunoblotting

MSC (5 × 10^4^ cells per well) were cultured in a 6‐well plate for 5 days before exposure to 0.6 mM of DETANONOate. At set time points (2, 6, and 24 hours), cells were washed and lysed using universal lysis buffer (Millipore). A Qubit Fluorometer and Quant‐iT Protein assay kit (Invitrogen) were used according to manufacturer's instructions to ensure equal loading of samples. Western blot and dot blot analysis were performed as previously described 32. In brief, protein lysates were denatured at 95°C and run on Tris‐HCl 10%–20% ready gels (Bio‐Rad) for Western blot or diluted in Tris‐buffered saline (TBS, Biorad USA) and added to the Bio‐Dot Microfiltration apparatus containing a prewet nitrocellulose membrane (Bio‐Rad). After transfer to nitrocellulose membrane and blocking in 5% BSA (Sigma) or 5% of milk in TBS‐Tween for 1 hour, membranes were incubated overnight in primary antibody at 4°C. Antibodies used were mouse anti‐SOD1 (1:2,000, R&D USA), mouse anti‐GSTP1 (1:2,000; Santa Cruz USA), rabbit anti‐peroxisome proliferator‐activated receptor‐gamma coactivator‐1alpha (PGC1α) (1:3,000; Santa Cruz), rabbit anti‐nuclear factor erythroid 2‐related factor 2 (Nrf2) (1:3,000; Santa Cruz), rabbit anticatalase (1:5,000; Abcam UK), mouse anti‐glyceraldehyde 3‐phosphate dehydrogenase (GAPDH) (1:5,000; Abcam), and antiactin (1:5,000; Abcam). Specific protein expression patterns were visualized by chemiluminescence using ECL Plus Western Blotting Detection System (Amersham USA). After developing, the ChemiDoc MP Imager (Biorad), Image Lab software was used to measure the integrated density. Values are expressed relative to loading control proteins GAPDH or actin. Western blots were used to confirm antibody specificity and for baseline comparison of protein expression between C‐MSC and MS‐MSC. Given the number of replicates over multiple time points, dot blots were used for determination of protein expression in experiments using exposure to DETANONOate.

### Real‐Time Polymerase Chain Reaction

RNA was extracted and cDNA produced using the Taqman gene expression cells‐Ct‐kit (Applied Biosystems USA) according to the manufacturer's instructions. RNA samples were quantified using a Quant‐iT RNA assay kit (Invitrogen) according to manufacturer's instructions to ensure equal loading. Real‐time polymerase chain reaction (RT‐PCR) was performed using the StepOnePlus Real‐Time PCR System (Applied Biosystems) with Assay‐on‐demand Gene Expression Products for SOD1, GSTP1, PGC1α, Nrf2, and GAPDH (Taqman MGB probe, FAM dye‐labeled, Applied Biosystems) using 10 ng cDNA in 20 μl of FAST master mix (Applied Biosystems). Reactions were run at 50°C for 2 minutes, 95°C for 20 seconds, 40 cycles of 95°C for 1 second, and 60°C for 20 seconds. Samples were analyzed in triplicate. Relative gene expression (relative quantification value) was calculated using the 2^−ΔΔCt^ method with GAPDH as reference (housekeeping) gene.

### SOD and GST Activity Assay

SOD and GST activities were quantified in cell lysates using the relevant colorimetric assay (Abcam) according to manufacturer's protocol. OD was measured at 450 nm for SOD and 340 nm for GST in kinetic mode.

### Statistical Analysis

All graphs were generated using GraphPad PRISM 5 (Graph Pad Software USA), which was also used for statistical analyses (*) other than those which used multiple regression (#). Where stated, a regression model which allowed for correlation between replicates isolated from the same individual (cluster option) was used for multivariant analyses including, where relevant, age, birth sex, presence of progressive MS, and time (STATA v12, StataCorp) USA. Nonparametric bootstrap analysis was used to estimate SE and confidence intervals (CI) to account for possible non‐normality of the parameter's distribution. Grouped analyses show mean ± SEM, and regression lines were fitted with 95% CI. Analyses were two sided and values of *p* < .05 considered statistically significant. Where regression was used, only those analyses where the 95% CI did not include zero were considered significant.

## Results

### Reduced Secretion of SOD1 and GSTP by MS‐MSC Associated with Duration of Progressive MS

**Figure 1:**
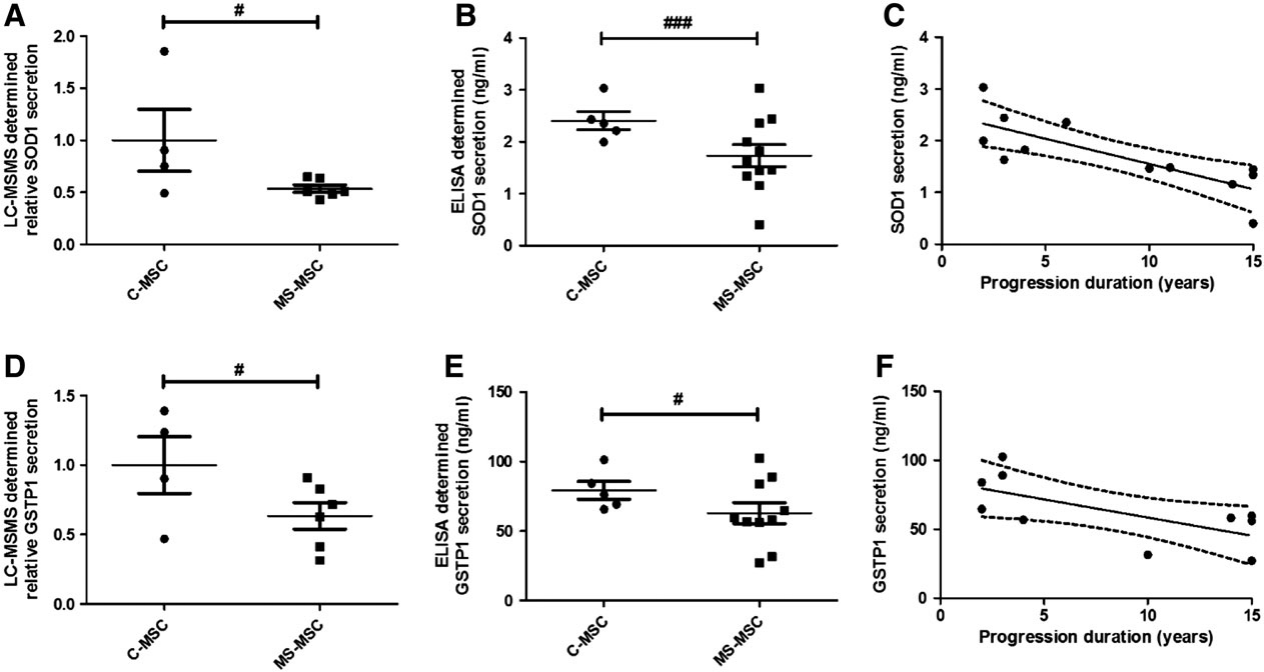
Reduced MS‐MSC secretion of SOD1 and GSTP1 in association with duration of progressive phase of MS. Analysis of the MSC secretome was undertaken using LC‐MSMS (C‐MSC *n* = 4; MS‐MSC *n* = 6) and ELISA (C‐MSC *n* = 5; MS‐MSC *n* = 11). **(A):** When the difference in age between the cohorts was accounted for, an independent, statistically significant effect of the presence of MS was seen with reduced secretion of SOD1 (regression analysis, #, *p* = .042, CI −0.093 to 3.9) as measured by LC‐MSMS. **(B):** Reduced secretion of SOD1 by MS‐MSC as determined by ELISA (regression analysis, ###, *p* < .001, CI −1.554 to −0.451). **(C):** SOD1 secretion by MS‐MSC as determined by ELISA negatively correlated with duration of the progressive phase of MS (Pearson *r* −.789, *p* = .002, CI −0.938 to −0.392). **(D):** Reduction in secretion of GSTP1 by MS‐MSC as determined by LC‐MSMS (regression analysis, #, *p* = .034, CI −0.975 to −0.039). **(E):** Reduction in GSTP1 in MS‐MSC measured by ELISA (regression analysis, #, *p* = .04, CI −45.332 to −1.021). **(F):** Negative correlation of GSTP1 secretion by MS‐MSC with duration of progressive disease (Pearson *r* −.665, *p* = .036, CI −0.913 to −0.061). The data are the means ± SEM of multiple biological replicates as listed. Abbreviations: CI, confidence interval; C‐MSC, control mesenchymal stromal cells; ELISA, enzyme‐linked immunosorbent assay; GSTP1, Glutathione S‐Transferase Pi 1; LC‐MSMS, liquid chromatography‐tandem mass spectrometry; MS, multiple sclerosis; MSC, mesenchymal stromal cells; MS‐MSC, mesenchymal stromal cells from patients with multiple sclerosis; SOD1, superoxide dismutase 1.

Using LC‐MSMS, SOD1, and GSTP1 were detected in the secretome of C‐MSC and MS‐MSC but there was a relative reduction in the secretion of both antioxidants by MS‐MSC (Fig. 1A, 1D). The results were confirmed using ELISA; an independent negative effect of the presence of progressive MS was seen on secretion of both SOD1 and GSTP1 when the effect of age was taken into consideration (Fig. 1B, 1E). Secretion of both SOD1 and GSTP1 correlated negatively with duration of the progressive phase of MS (Fig. 1C, 1F). Catalase secretion was not detected in the MSC secretome using LC‐MSMS.

### Reduced Expression of SOD1 and GSTP with Reduced Activity of SOD and GST in MS‐MSC

**Figure 2:**
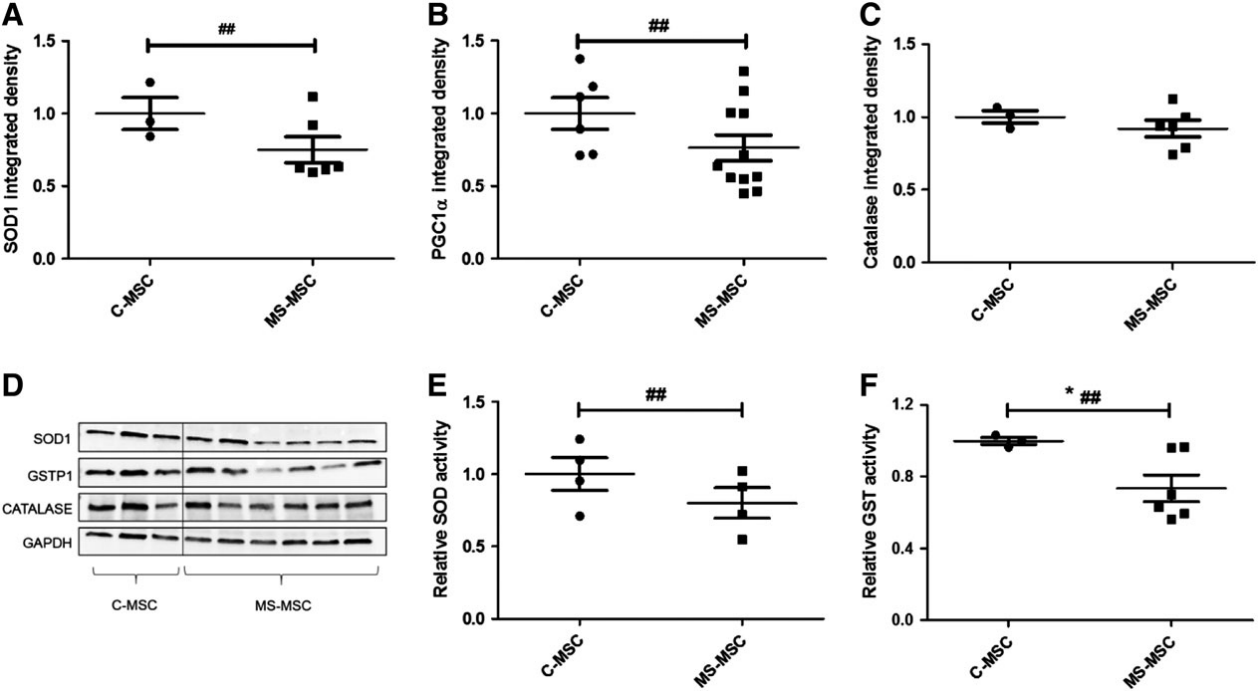
MSC expression and activity of antioxidants in standard culture conditions. Densitometric analysis of Western blots was used to examine protein expression in standard culture conditions (C‐MSC *n* = 3; MS‐MSC *n* = 7). **(A):** SOD1 expression was reduced in MS‐MSC compared to C‐MSC (regression analysis, ##, *p* = .002, CI −0.737 to −0.159). **(B):** GSTP1 expression was reduced in MS‐MSC (regression analysis, ##, *p* = .006, CI −1.39 to −0.23). **(C):** Catalase expression was unaltered. **(D):** Representative bands are shown. **(E):** SOD activity was reduced in MS‐MSC (*n* = 4) compared to C‐MSC (*n* = 4; regression analysis, ##, *p* = .003, CI −25.706 to −5.288). **(F):** GST activity was reduced in MS‐MSC (*n* = 6) compared to C‐MSC (*n* = 3; Mann Whitney test, *, *p* = .038; regression analysis, ##, *p* = .006, CI −0.854 to −0.14). The data are the means ± SEM of multiple biological replicates as listed. Abbreviations: CI, confidence interval; C‐MSC, control mesenchymal stromal cells; GAPDH, glyceraldehyde‐3‐phosphate dehydrogenase; GSTP1, Glutathione S‐Transferase Pi 1; MS, multiple sclerosis; MSC, mesenchymal stromal cell; MS‐MSC, mesenchymal stromal cells from patients with multiple sclerosis; SOD1, superoxide dismutase 1.

Immunoblotting was used to examine the expression of SOD1 and GSTP1 and demonstrated reduced expression in MS‐MSC when adjustment was made for the effect of age (Fig. 2A, 2B). There was no difference in catalase expression (Fig. 2C), and additional analyses of catalase activity were not therefore undertaken. Antioxidant activity was assessed using activity assays for total SOD and GST attributable to the lack of availability of an assay capable of differentiating between activity of specific isoforms. Accounting for effects of age, MS‐MSC had reduced activity of both SOD (Fig. 2E) and GST (Fig. 2F).

### MS‐MSC have Increased In Vitro Susceptibility to Nitrosative Stress

C‐MSC was exposed to DETANONOate at varying concentrations for 24 hours, and cell survival was determined using MTT assay. Based on our previous experience with in vitro assays of cell toxicity and survival, we selected the dose for subsequent cell survival studies as that inducing approximately 33% cell death (0.6 mM DETANONOate inducing 37% cell survival; data not shown).

**Figure 3:**
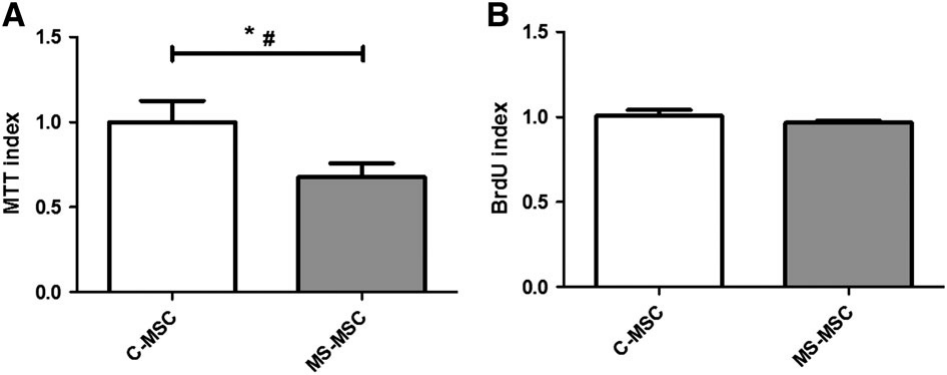
MS‐MSC show increased susceptibility to DETANONOate in vitro. **(A):** MSC were exposed to 0.6 mM DETANONOate for 24 hours following which viability was measured using the MTT assay. Relative to C‐MSC (*n* = 7), MS‐MSC (*n* = 13) were more vulnerable to DETANONOate‐induced toxicity and a significant effect was also seen when the potential confounding effect of age was taken into consideration (Mann Whitney test, *, *p* < .05; regression analysis, #, *p* = .04, CI −0.801 to −0.016). **(B):** Difference in MTT signal cannot be attributed to altered proliferation rates as changes in BrdU expression were not observed, including when age was accounted for (C‐MSC *n* = 3 and MS‐MSC *n* = 3). The data are the means ± SEM of multiple biological replicates as listed. Abbreviations: CI, confidence interval; BrdU, bromodeoxyuridine; C‐MSC, control mesenchymal stromal cells; MS‐MSC, mesenchymal stromal cells from patients with multiple sclerosis; MTT, methylthiazolyldiphenyl‐tetrazolium bromide.

C‐MSC and MS‐MSC were exposed to 0.6 mM DETANONOate for 24 hours, and the effects on cell survival are compared. MS‐MSC displayed increased susceptibility to DETANONOate, an effect sustained statistically when regression analysis was undertaken to account for effect of age (Fig. 3A). No differences in MS‐MSC survival were observed according to MS subtype (primary or secondary progressive disease) or duration of progressive phase of the disease.

Despite the short duration of experiments, BrdU was used to exclude the possibility that differences in cell survival between populations of C‐MSC and MS‐MSC could be explained by altered proliferation rate; no difference in MSC proliferation between MS and control cohorts was noted following exposure of MSC to 6 mM DETANONOate for 24 hours (Fig. 3B).

### DETANONOate Induces Accelerated Senescence in MS‐MSC

**Figure 4:**
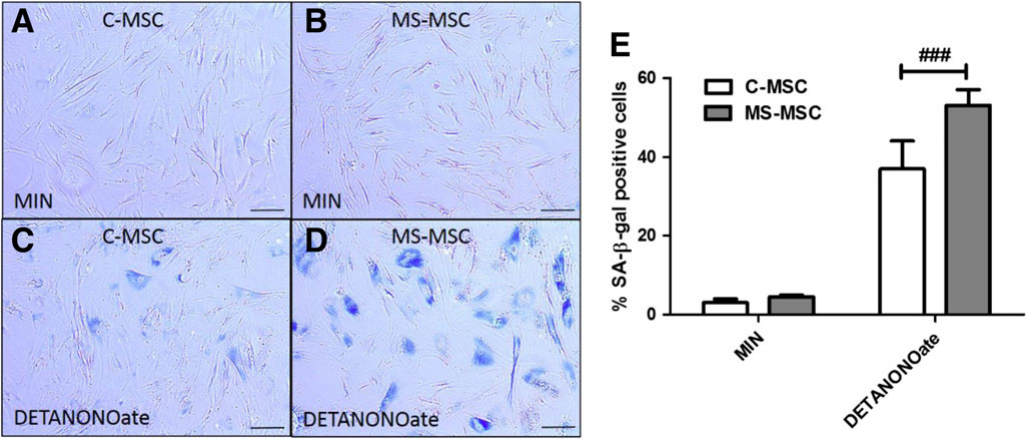
Increased susceptibility of MS‐MSC to senescence following DETANONOate exposure. **(A):** C‐MSC under standard culture conditions show little or no expression of SA‐β‐gal (blue). **(B):** MS‐MSC also have few SA‐β‐gal positive cells at low passage number under standard culture conditions. **(C):** Following exposure to DETANONOate (0.6 mM for 24 hours), C‐MSC show increased expression of SA‐β‐gal at 5 days. **(D):** MS‐MSC show disproportionally greater expression of SA‐β‐gal following DETANONOate exposure indicative of increased senescence. **(E):** When effects of age are taken into consideration, MS‐MSC show higher expression of SA‐β‐gal expression in response to DETANONOate exposure (regression analysis, ###, *p* < .001, CI 13.125 to 36.184). The data are the means ± SEM of multiple biological replicates (C‐MSC (*n* = 3) and MS‐MSC (*n* = 4); Scale bar 100 μm. Abbreviations: CI, confidence interval; C‐MSC, control mesenchymal stromal cells; MIN, minimal medium; MS‐MSC, mesenchymal stromal cells from patients with multiple sclerosis; SA‐β‐gal, senescence‐associated β‐galactosidase.

Increasing MSC donor age is known to increase senescence 17, and multiple regression analysis was used in all analyses of SA‐β‐gal expression. C‐MSC and MS‐MSC at p4 were treated with 0.6 mM DETANONOate for 24 hours following which the SA‐β‐gal assay was performed to quantify senescent cells (blue staining). Very low levels of SA‐β‐gal expression are seen following MSC exposure to MIN alone for 24 hours (Fig. 4A, 4B). As expected, MSC SA‐β‐gal expression increased following DETANONOate exposure consistent with an increase in number of senescent MSC (Fig. 4C–4E). Senescent cells displayed expected phenotypical changes including enlarged flattened morphology, granular cytoplasm, vacuoles and enlarged nuclei (Fig. 4C, 4D). Following DETANONOate exposure, the proportion of senescent cells as defined by those expressing SA‐β‐gal was significantly greater in MS‐MSC cultures compared with the proportion of senescent C‐MSC (Fig. 4E).

### Reduced MS‐MSC Expression of SOD1 and GSTP1 Following Exposure to Nitrosative Stress

**Figure 5:**
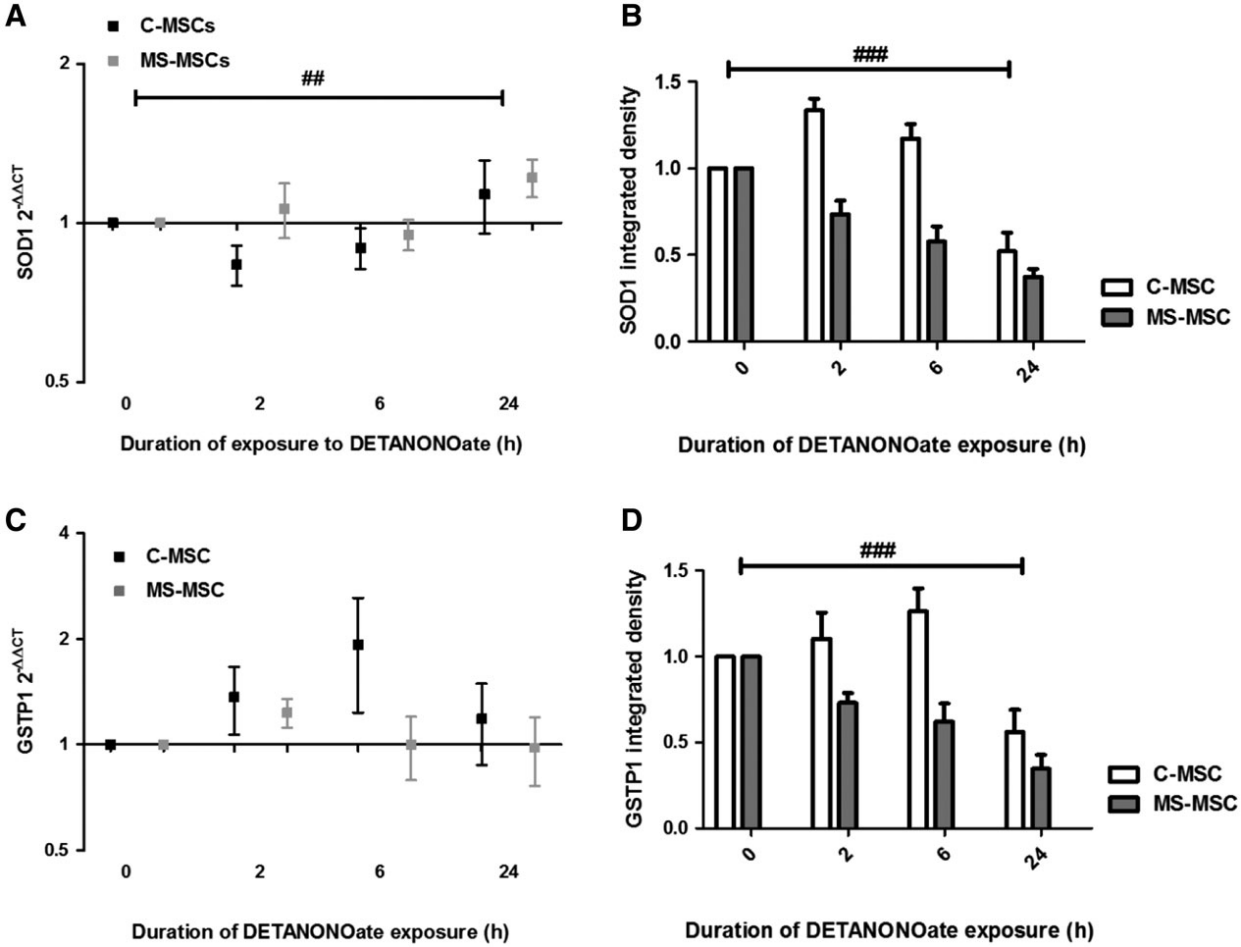
Reduced MS‐MSC expression of SOD1 and GSTP1 under conditions of nitrosative stress. Following exposure to DETANONOate, SOD1 and GSTP1 gene and protein expression relative to baseline were examined over a 24‐hour period. **(A):** *SOD1* expression, measured by RT‐PCR relative to GAPDH, increased over time with exposure to DETANONOate in both C‐MSC and MS‐MSC (regression analysis, ##, *p* = .008, CI 0.002 to 0.016). There was no difference between *SOD1* expression in C‐MSC and MS‐MSC at any time point examined. **(B):** Protein expression of SOD1 in response to nitrostative stress in C‐MSC and MS‐MSC was examined using immunoblotting. Following adjustment for age, SOD1 expression declined with exposure to DETANONOate (regression analysis, ###, *p* < .001, CI −0.027 to −0.019). An independent, negative effect on SOD1 expression attributable to progressive MS was seen following adjustment for age (regression analysis, *p* < .001, CI −0.515 to −0.226). **(C):** A significant change in *GSTP1* expression was not observed in either C‐MSC or MS‐MSC following exposure to DETANONOate including after correction for age. **(D):** Overall, GSTP1 protein expression following exposure to DETANONOate fell in both C‐MSC and MS‐MSC and an independent negative effect of progressive MS was observed (regression analysis, ###, *p* < .001, CI −0.47 to −0.326). The data are the means ± SEM of multiple biological replicates C‐MSC *n* = 3, MS‐MSC *n* = 4. Abbreviations: CI, confidence interval; C‐MSC, control mesenchymal stromal cells; GAPDH, glyceraldehyde‐3‐phosphate dehydrogenase; GSTP1, Glutathione S‐Transferase Pi 1; MS, multiple sclerosis; MS‐MSC, mesenchymal stromal cells from patients with multiple sclerosis; RT‐PCR, real time polymerase chain reaction; SOD1, superoxide dismutase 1.

Expression of SOD1 and GSTP1 under conditions of nitrosative stress were examined following exposure of MSC to DETANONOate for 24 hours as previously. *SOD1* gene expression increased after 24 hours of DETANONOate exposure and no difference in response was seen between C‐MSC and MS‐MSC (Fig. 5A). However, protein expression of SOD1 declined in both C‐MSC and MS‐MSC with an earlier and greater decline observed in MS‐MSC (Fig. 5B). No significant changes in *GSTP1* gene expression were seen at any of the time points examined in C‐MSC or MS‐MSC (Fig. 5C) although GSTP1 protein expression was lower in MS‐MSC at 2 and 6 hours and, adjusting for age, an independent, negative, statistically significant effect of the presence of MS on GSTP1 expression was observed (Fig. 5D).

### Reduced Nrf2 Expression in MS‐MSC in Standard Culture Conditions and Following Nitrosative Stress

**Figure 6:**
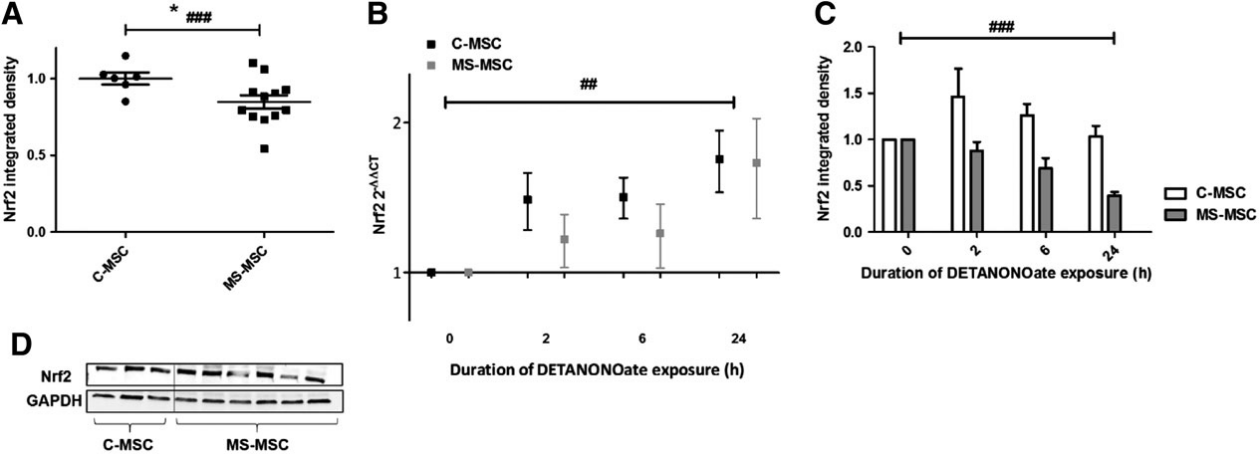
Reduced Nrf*2* expression in MS‐MSC in standard culture conditions and in response to DETANONOate exposure. **(A):** Reduced expression of Nrf2 in MS‐MSC (*n* = 12) compared with C‐MSC (*n* = 6) in standard culture conditions as determined by Western blot densitometric analysis (Student's *t* test * *p* = .04; regression analysis, ###, *p* < .001, CI −0.897 to −0.293). **(B):** Representative bands for Nrf2 and GAPDH are demonstrated. **(C):** *Nrf2* expression in C‐MSC and MS‐MSC at baseline and following exposure to DETANONOate was examined over a 24‐hour period using RT‐PCR and the fold difference relative to the average expression in cultures not exposed to DETANONOate was calculated by the (2^−ΔΔCT^) method with GAPDH as the calibrator. Expression increased with time of exposure (C‐MSC *n* = 3, MS‐MSC *n* = 4; regression analysis, ##, *p* = .007, CI −0.011 to −0.002) but, although expression of *Nrf2* was lower in MS‐MSC at all time points examined, this did not reach statistical significance. **(D):** Expression of Nrf2 by C‐MSC (*n* = 3) and MS‐MSC (*n* = 7) following exposure to DETANONOate was examined using immunoblotting; overall, a negative effect of DETANONOate exposure on Nrf2 expression was seen over time (regression analysis, ###, *p* < .0001, CI −0.024 to 0.0123) with an independent, negative effect of the presence of progressive MS was seen following adjustments for age (regression analysis, *p* < .0001, CI −0.708 to −0.336). The data are the means ± SEM of multiple biological replicates as listed. Abbreviations: CI, confidence interval; C‐MSC, control mesenchymal stromal cells; GAPDH, glyceraldehyde‐3‐phosphate dehydrogenase; MS, multiple sclerosis; MS‐MSC, mesenchymal stromal cells from patients with multiple sclerosis; Nrf2, nuclear factor erythroid 2‐related factor 2; RT‐PCR, real‐time polymerase chain reaction.

The transcriptional factor Nrf2 is a key regulator of antioxidant‐enzyme genes, and we therefore explored (i) whether MS‐MSC have reduced expression of Nrf2 under standard culture conditions and (ii) whether Nrf2 expression increases in response to DETANONOate exposure. Western blot analysis under standard culture conditions demonstrated that MS‐MSC express lower levels of Nrf2 compared to C‐MSC (Fig. 6A). *Nrf2* gene expression was upregulated in both C‐MSC and MS‐MSC over the 24‐hour period following exposure to DETANONOate (Fig. 6C). Following exposure of C‐MSC to nitrosative stress, Nrf2 protein expression showed a trend toward an increase in expression, particularly after 2 and 6 hours of DETANONOate exposure (Fig. 6D). In contrast, MS‐MSC expression of Nrf2 declined over the 24‐hour period following exposure to DETANONOate (Fig. 6D).

### MS‐MSC in Standard Culture Conditions and Following Nitrosative Stress Have Reduced Expression of PGC1α

**Figure 7:**
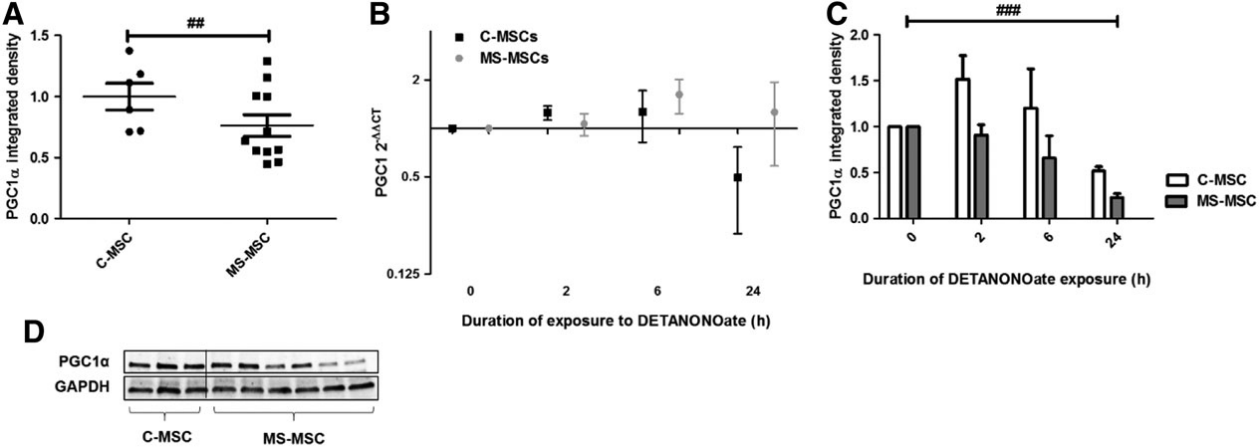
Reduced PGC1α expression in MS‐MSC in standard culture conditions and in response to DETANONOate exposure. (A): Densitometric analysis of Western blots demonstrated reduced PGC1α protein expression in MS‐MSC (*n* = 12) compared to C‐MSC (*n* = 6), and this reached statistical significance following adjustment for age (Student's *t* test *p* = .09; regression analysis, ##, *p* = .009, CI −0.911 to −0.127). **(B):** Representative bands for PGC1α and GAPDH are shown. **(C):** *PGC1α* gene expression following DETANONOate exposure relative to baseline expression was examined over a 24‐hour period using RT‐PCR in both C‐MSC (*n* = 3) and MS‐MSC (*n* = 4). Using GAPDH as the calibrator, no change in *PGC1α* expression was seen in either C‐MSC and MS‐MSC, including when analysis was undertaken to account for the effects of age. **(D):** Using immunoblotting, the change in PGC1α protein expression in response to DETANONOate exposure was examined in C‐MSC (*n* = 3) and MS‐MSC (*n* = 7). Overall, reduced PGC1α expression with increasing time of exposure to DETANONOate was observed (regression analysis, ###, *p* < .001, CI −0.482 to −0.042). An independent, negative effect of the presence of MS was seen following adjustment for age (regression analysis, *p* = .027, CI −0.91 to 0.054). The data are the means ± SEM of multiple biological replicates as listed. Abbreviations: CI, confidence interval; C‐MSC, control mesenchymal stromal cells; GAPDH, glyceraldehyde‐3‐phosphate dehydrogenase; MS, multiple sclerosis; MS‐MSC, mesenchymal stromal cells from patients with multiple sclerosis; PGC1α, peroxisome proliferator‐activated receptor‐gamma coactivator‐1alpha; RT‐PCR, real time polymerase chain reaction.

The master regulator of ROS‐detoxifying enzymes PGC1α was measured in MSC from both control subjects and patients with MS under standard culture conditions and after 2, 6, and 24 hours of DETANONOate exposure. Protein expression of PGC1α was significantly decreased in MS‐MSC compared to C‐MSC under standard culture conditions following adjustment for age (Fig. 7A). At the time points examined after DETANONOate treatment (2, 6, and 24 hours), there was no change in *PGC1α* gene expression in either C‐MSC or MS‐MSC (Fig. 7C). However, C‐MSC and MS‐MSC showed differing responses to DETANONOate exposure in the level of PGC1α protein expression with a reduction observed in MS‐MSC (Fig. 7D).

## Discussion

MSC have been identified as an excellent candidate for cell therapy in a wide variety of clinical contexts. Increasingly, however, attention is being given to the quality of MSC used for therapy, particularly autologous cells which have been exposed to disease microenvironment. Detailed examination of MSC isolated from donors with chronic diseases is yielding novel insights into disease pathophysiology and in vivo MSC function. In this study, we have examined the susceptibility of MS‐MSC to nitrosative stress in vitro together with an analysis of expression and activity of key antioxidants and their regulators under basal cell culture conditions and in response to nitrosative stress.

We have demonstrated that nitrosative stress differentially affects MS‐MSC, inducing changes of accelerated senescence. In basal cell culture conditions and following exposure to nitrosative stress, MS‐MSC have reduced levels of expression of Nrf2 and PGC1α known regulators of the antioxidant response. There is concomitant reduced expression of antioxidants SOD1 and GSTP although catalase expression was unaltered. In vitro activity of SOD and GST was both reduced in MS‐MSC.

PGC1α has a key role in mitochondrial biogenesis, respiration, and induction of antioxidant programs 33. Reduced expression in MS has been noted and is thought to contribute to mitochondrial changes and neuronal loss 34. PGC1α lacks DNA‐binding activity but interacts with and coactivates several transcription factors including Nrf2, which controls constitutive and inducible expression of an array of antioxidant enzymes including SOD, GST, and catalase 35. Downregulation of *PGC1α* has been shown to affect the antioxidant response in Friedreich's ataxia 36 and expression of *PGC1α* has been noted to be lower in adipose tissue‐derived MSC from elderly donors 37. Interestingly, MSC isolated from patients with PSP had fivefold lower levels of expression of *PGC1α* compared with control MSC 19 and, in an induced pluripotent stem cell model of Parkinson's disease, S‐nitrosylation of the transcription factor myocyte enhancer factor 2c (MEF2C) contributes to mitochondrial dysfunction and apoptotic cell death via dysregulation of the PGC1α‐MEF2C transcriptional network 38, a mechanism which may have contributed to the decrease in PGC1α expression seen in MS‐MSC at baseline and in response to nitrosative stress.

Nrf2 is a regulator of cellular resistance to oxidative stress with an integral role in controlling expression of antioxidant response element‐dependent genes. Nrf2 is known to be activated by nitrosative agents 39, and its repression has been associated with ageing 35 including in a model of Hutchinson‐Gilford progeria syndrome where MSC attrition is accelerated 40. Nrf2 has been shown to maintain the self‐renewal potential of MSC 41, and overexpression induces MSC proliferation and reduces apoptosis, including in response to oxidative stress 42, 43. In models of MS, loss of Nrf2 has been shown to exacerbate neurological deficits 44, 45 with activation being protective 46. Dimethyl fumarate, a disease modifying agent for the treatment of MS, may act at least in part via activation of the Nrf2 pathway 47, 48.

The mechanism(s) underlying reduced expression of Nrf2 in MS‐MSC in response to nitrostative stress has not been identified to date. Regulation of Nrf2 is complex and involves multiple pathways but a major factor is known to be the variation in the level of protein stability; under basal conditions, Nrf2 is rapidly degraded by the 26S proteasome, and this allows for an immediate increase when ubiquitylation and proteasomal degradation are inhibited by stimuli including redox stress 49. Low levels of Nrf2 in the absence of environmental stressors have also been attributed to translational repression 50, suggesting that a possible alternative or additional mechanism for the failure to increase Nrf2 expression in MS‐MSC was failure to reverse the translational repression of Nrf2 appropriate for basal conditions. Indeed, we note that in experimental allergic encephalitis as in our experiments, Nrf2 mRNA levels are maintained but expression is reduced suggesting either impaired translation or changes in post‐translational processing 51.

We note the differences in age between the control and MS cohorts and have used a multiple regression model to account for this. There was no sex bias in the cohorts, and an independent effect attributable to birth sex was not observed in the regression analyses. In addition to age‐mismatch between cohorts, an additional potential confounding factor is the difference in MSC source between patients and control subjects, that is, marrow from the posterior iliac crest and femoral head, respectively. However, pelvic marrow is generally taken to be the gold standard for MSC isolation 52 and while the indication for hip replacement in the control cohort could be a possible additional confounding factor, MSC changes in the context of osteoarthritis have not been consistently reported 53, 54, 55, 56. None of the control subjects or those with primary progressive MS had exposure to immunomodulatory drugs or DMT. We were unable to perform regression analyses to determine if there was an independent effect of prior exposure to DMT in the cohort with secondary progressive MS attributable to the low numbers of patients included in each analysis. However, there was no differential effect of disease subtype on any of the analyses undertaken and this, combined with the time interval between exposure and marrow collection, would make it relatively unlikely that our disease‐specific results can be explained by DMT exposure.

Our findings are consistent with a failure of homeostasis in MS‐MSC attributable to dysregulation of PGC1α and Nrf2‐mediated antioxidant responses, which in turn contribute to a phenotype of premature aging. We note that a similar mechanism has been proposed in the context of vascular oxidative stress associated with ageing 57. The negative association between antioxidant responses (SOD1 and GSTP1 secretion) and duration of progressive MS raises the possibility that chronic exposure to disease adversely affects MSC function with functional consequences for the bone marrow microenvironment including, for example, alterations in regulatory and pro‐inflammatory T‐cell populations. While this may, in turn, contribute to disease pathophysiology, the finding adds to our previous work, demonstrating changes consistent with accelerated ageing in MS bone marrow and has clear implications for both MSC‐based and autologous haematopoietic stem cell therapy for MS and other conditions where oxidative stress plays a role in disease 17. It would suggest that bone marrow‐derived cell therapy is more likely to be effective in MS patients with a shorter phase of progressive disease although, based on currently available data, we are unable to speculate regarding a cut‐off in terms of age or disease duration for consideration of therapy.

Future work will include analyses of expression of additional Nrf2 and PGC1α target genes, examination of vascular oxidative stress associated with MS, exploration of whether reversal of the identified deficits in antioxidant response of MS‐MSC improves the neuroprotective potential of MS‐MSC, and further analysis of bone marrow microenvironment and function in MS.

## Conclusion

We have demonstrated that MS‐MSC have reduced expression of Nrf2 and PGC1α under basal culture conditions with reduced expression of key antioxidants SODs and GSTP and reduced activity of SOD and GST. Furthermore, MS‐MSC have increased susceptibility to nitrosative stress which is associated with reduced expression of Nrf2 and PGC1α. Our findings have significant implication for those developing autologous MSC‐based therapies for MS as identification and correction of the factors responsible are likely to be required if the full potential of MSC for autologous cell‐based treatment is to be realized. Furthermore, we predict that understanding the mechanisms involved will yield novel insights into the pathophysiology of MS and aid identification of new drug targets for the treatment of progressive MS.

## Authors Contribution

J.R.: conception and design, collection of data, data analysis and interpretation, manuscript writing, final approval of manuscript; P.S.: provision of study material or patients, final approval of manuscript; K.K.: data analysis and interpretation, final approval of manuscript; K.J.H.: collection of data, final approval of manuscript; A.W.: data analysis interpretation, final approval of manuscript; N.J.S.: conception and design, manuscript writing, final approval of manuscript; C.M.R.: conception and design, data analysis and interpretation, manuscript writing, final approval of manuscript.

## Disclosure of Potential Conflicts of Interest

The authors indicated no potential conflicts of interests.

## Supporting information


**Supporting Information Table 1** Summary data for both control and MS cohorts including information regarding which samples were included for each of the analyses undertaken and history of exposure to disease modifying drugs (DMT)
**Supporting Information Table 2** Eligibility criteria for the ‘ACTiMuS’ trial
