# Supplementary material for: Dysregulation of Mesenchymal Stromal Cell Antioxidant Responses in Progressive Multiple Sclerosis
Source: Stem Cells Transl Med. 2018 Jul 31;7(10):748–58. doi: 10.1002/sctm.18-0045 (PMC6186266; doi:10.1002/sctm.18-0045)
Supplement: Supplementary file 1 — Supporting Information Table 1 Summary data for both control and MS cohorts including information regarding which samples were included for each of the analyses undertaken and history of exposure to disease modifying drugs (DMT) Supporting Information Table 2 Eligibility criteria for the ‘ACTiMuS’ trial [file SCT3-7-748-s001.docx]

**Table 1** Summary data for both control and MS cohorts including information regarding which samples were included for each of the analyses undertaken and history of exposure to disease modifying drugs (DMT)

| **Patient** | **Sex** | **Age** | **Classification MS** | **Duration progression (years)** | **Experiments performed** | **DMT** |
| --- | --- | --- | --- | --- | --- | --- |
| Control 1 | F | 65 | n/a | n/a | WB | n/a |
| Control 2 | M | 54 | n/a | n/a | ELISA | n/a |
| Control 3 | M | 66 | n/a | n/a | WB; ELISA | n/a |
| Control 4 | M | 49 | n/a | n/a | ELISA; SOD ACTIVITY ASSAY | n/a |
| Control 5 | F | 60 | n/a | n/a | WB; ELISA | n/a |
| Control 6 | M | 58 | n/a | n/a | WB; ELISA; BGAL; MTT; BrdU | n/a |
| Control 7 | F | 48 | n/a | n/a | MTT | n/a |
| Control 8 | M | 71 | n/a | n/a | MTT | n/a |
| Control 9 | F | 53 | n/a | n/a | MTT; WB; ELISA; GST ACTIVITY ASSAY | n/a |
| Control 10 | F | 68 | n/a | n/a | MTT; DOT BLOT; PCR; BGAL; WB, ELISA; SOD ACTIVITY ASSAY; BrdU | n/a |
| Control 11 | F | 55 | n/a | n/a | MTT; DOT BLOT; PCR; BGAL; WB; SOD and GST ACTIVITY ASSAY, BrdU | n/a |
| Control 12 | M | 58 | n/a | n/a | MTT; DOT BLOT; PCR; BGAL; SOD and GST ACTIVITY ASSAY | n/a |
| Control 13 | F | 83 | n/a | n/a | MTT | n/a |
| Control 14 | M | 59 | n/a | n/a | WB; ELISA | n/a |
| **Mean (yrs)** |  | **60.5** |  |  |  |  |
| **Median (yrs)** |  | **58.5** |  |  |  |  |
|  |  |  |  |  |  |  |
| MS 1 | M | 48 | PP | 4 | MTT; DOT BLOT; BGAL; WB; ELISA; SOD ACTIVITY ASSAY; BrdU | none |
| MS 2 | F | 48 | PP | 15 | ELISA | none |
| MS 3 | M | 60 | SP | 10 | ELISA | none |
| MS 4 | M | 33 | SP | 3 | WB; ELISA | Beta-interferon |
| MS 5 | F | 47 | PP | 6 | WB; ELISA | none |
| MS 6 | F | 59 | SP | 15 | WB; ELISA | none |
| MS 7 | M | 55 | SP | 2 | WB; ELISA | none |
| MS 8 | M | 56 | SP | 15 | MTT; DOT BLOT; BGAL; WB; ELISA | none |
| MS 9 | F | 53 | SP | 3 | WB; ELISA | none |
| MS 10 | M | 49 | PP | 14 | WB; ELISA | none |
| MS 11 | M | 64 | PP | 15 | MTT; DOT BLOT; PCR; WB; ELISA; BrdU | none |
| MS 12 | M | 50 | PP | 2 | WB; ELISA | none |
| MS 13 | M | 63 | SP | 12 | MTT; DOT BLOT; BGAL; SOD ACTIVITY ASSAY | Beta-interferon |
| MS 14 | F | 41 | SP | 2 | MTT; DOT BLOT; PCR; WB | Glatiramer |
| MS 15 | F | 50 | PP | 4 | MTT | none |
| MS 16 | F | 49 | SP | 4 | MTT; DOT BLOT; BGAL; PCR; WB; SOD ACTIVITY ASSAY; BrdU, GST ACTIVITY ASSAY | Beta-interferon |
| MS 17 | F | 58 | PP | 10 | WB | none |
| MS 18 | F | 57 | SP | 3 | MTT | Glatiramer |
| MS 19 | F | 54 | SP | 2 | MTT | Beta-interferon, glatiramer, alemtuzumab |
| MS 20 | F | 54 | PP | 4 | MTT | none |
| MS 21 | M | 53 | PP | 20 | MTT | none |
| MS 22 | F | 52 | SP | 6 | MTT | none |
| MS 23 | F | 47 | PP | 17 | DOT BLOT; SOD ACTIVITY ASSAY | none |
| MS 24 | M | 53 | SP | 4 | MTT; PCR | none |
| MS 25 | M | 46 | PP | 13 | GST ACTIVITY ASSAY | none |
| MS 26 | F | 58 | PP | 10.5 | GST ACTIVITY ASSAY | none |
| MS 27 | F | 47 | SP | 3 | GST ACTIVITY ASSAY | none |
| MS 28 | F | 55 | SP | 15.5 | GST ACTIVITY ASSAY | Beta-interferon |
| MS 29 | M | 58 | SP | 2 | GST ACTIVITY ASSAY | Beta-interferon |
| **Mean (yrs)** |  | **52.31** |  |  |  |  |
| **Median (yrs)** |  | **53** |  |  |  |  |

**Table 2** Eligibility criteria for the ‘ACTiMuS’ trial

| **Inclusion criteria** | **Exclusion critieria** |
| --- | --- |
|  |  |
| Either sex, 18-65 years old  Diagnosis of clinically-definite MS as defined by the McDonald criteria  MS disease severity EDSS 4 – 6  Disease duration >5 years  Disease progression (not attributable to relapse) in the year prior to entry  Signed, written informed consent  Willing and able to comply with study visits according to protocol for the full study period | Pregnancy, breastfeeding or lactation  History of autologous/allogeneic bone marrow transplantation or peripheral blood stem cell transplant  Bone marrow insufficiency  History of lymphoproliferative disease or previous total lymphoid irradiation  Immune deficiency  History of current or recent (<5 years) malignancy  Chronic or frequent drug-resistant bacterial infections or presence of active infection requiring antimicrobial treatment  Frequent and/or serious viral infection  Systemic or invasive fungal disease within 2 years of entry to study  Significant renal, hepatic, cardiac or respiratory dysfunction  Contraindication to anaesthesia  Bleeding or clotting diathesis  Current or recent (within preceding 12 months) immunomodulatory therapy other than corticosteroid therapy  Treatment with corticosteroids within the preceding 3 months  Significant relapse within preceding 6 months  Predominantly relapsing-remitting disease over preceding 12 months  Radiation exposure in the past year other than chest / dental x-rays  Previous claustrophobia  The presence of any implanted metal or other contraindication to MRI  Participation in another experimental study or treatment within previous 24 months |
